# Oceanic SACZ produces an abnormally wet 2021/2022 rainy season in South America

**DOI:** 10.1038/s41598-023-28803-w

**Published:** 2023-01-26

**Authors:** Luciano P. Pezzi, Mario F. L. Quadro, Everaldo B. Souza, Arthur J. Miller, Vadlamudi B. Rao, Eliana B. Rosa, Marcelo F. Santini, Andréia Bender, Ronald B. Souza, Mylene J. Cabrera, Claudia K. Parise, Jonas T. Carvalho, Luciana S. Lima, Maria Rita L. de Quadros, Douglas M. Nehme, Jaime F. António

**Affiliations:** 1 Laboratory of Ocean and Atmosphere Studies (LOA), Earth Observation and Geoinformatics Division (DIOTG), National Institute for Space Research (INPE), São José dos Campos, São Paulo Brazil; 2 Federal Institute of Santa Catarina (IFSC), Florianópolis, Santa Catarina Brazil; 3 Federal University of Para (UFPA), Belém, Pará Brazil; 4 Scripps Institution of Oceanography (SIO), University of California San Diego (UCSD), San Diego, CA USA; 5 National Center for Monitoring and Early Warning of Natural Disasters (CEMADEN), São José dos Campos, São Paulo Brazil; 6 Earth System Numerical Modeling Division, National Institute of Space Research (INPE), Cachoeira Paulista, São Paulo Brazil; 7 Laboratory for Climate Studies and Modelling, Department of Oceanography, Federal University of Maranhão (UFMA), São Luiz, Maranhão Brazil; 8 Ocean Predictions and Applications, Centro Euro-Mediterraneo sui Cambiamenti Climatici, via Marco Biagi 5, 73100 Lecce, Italy; 9 Federal University of Rio de Janeiro (UFRJ), Rio de Janeiro, Rio de Janeiro Brazil

**Keywords:** Climate change, Ocean sciences, Physical oceanography, Atmospheric science, Atmospheric dynamics

## Abstract

The oceanic South Atlantic Convergence Zone (SACZ) has played a major role during South America’s 2021/2022 summer extreme rainy season, being responsible for more than 90% of the precipitation in some regions of Southeast Brazil and in some regions of the Southwestern Atlantic Ocean (SWA). The summer of 2021/2022 was unique and rare and considered an abnormally humid season as verified by official Brazilian Institutes. First, the unusual number of cases of SACZ episodes (seven), was the highest recorded in the last decade. Second, all the cases that occurred were oceanic SACZ that assumed characteristics of an Atmospheric River and produced an excessively anomalous amount of precipitation during this period. Excess precipitation along with the regions located in mountainous and very uneven relief, which by orographic effects favors high precipitation volumes, were responsible for amplifying the observed impacts, such as landslides and floods that caused several losses to society. We also showed the main effects of coupling and interaction between the waters of the surface layer of the SWA and the atmosphere. Our learning from this study ends with the unprecedented results of how the marine atmospheric boundary layer (MABL) is locally modulated by the sea surface temperature (SST) that lies just below it. Until the present moment, we emphasize that this important mechanism has not been widely highlighted in the literature, showing that even though the ocean is colder than before oceanic SACZ is established, it is still warmer than the overlying air, thus, the ocean continues to be an active source of heat and moisture for the atmosphere and enhances the MABL instability process.

## Introduction

**Figure 1:**
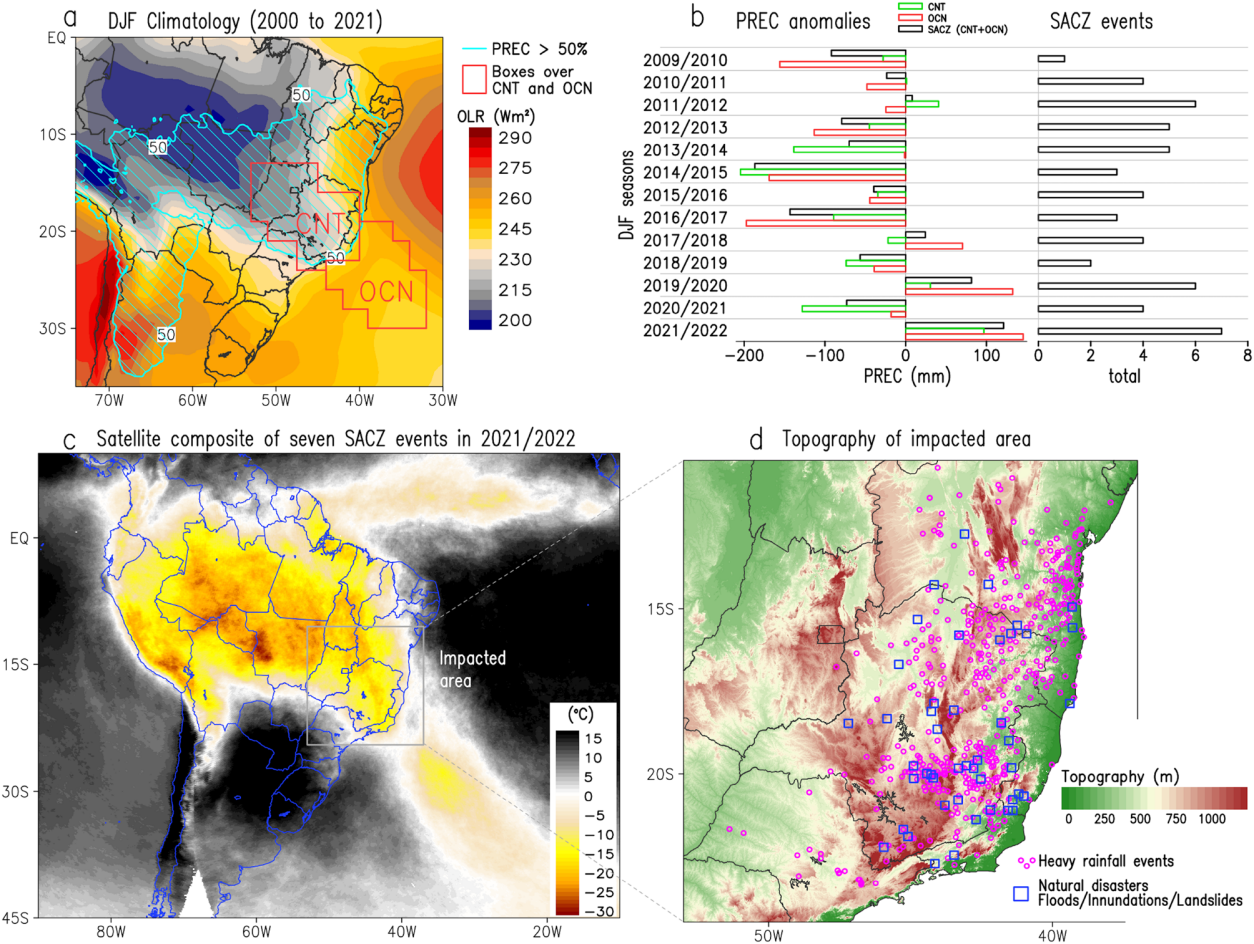
(**a**) December, January, and February (DJF) climatology for the period from 2000 to 2021 of Outgoing Longwave Radiation (OLR) shaded (W m^−2^) and percentage of precipitation (PREC) blue hatched (contours, %) emphasizing areas containing more than 50% of the annual total. (**b**) Time-series of box-averaged OLR, PREC anomalies over the continent (CNT), the ocean (OCN), and the whole SACZ area (CNT + OCN), and the total number of SACZ events (according to Rosa et al.^8^) during each DJF season. (**c**) Cloud top brightness temperature (°C) composite for the seven oceanic SACZ events in DJF 2021/2022. (**d**) Topography (shaded in m). The CNT and OCN areas are indicated in (**a**) as polygons in red lines. Symbols in (**d**) display heavy rainfall (purple circles) and natural disasters (blue squares) resulting from inundations, flash floods, and landslides. Grid Analysis and Display System (GrADS), Version 2.2.1.oga.1. http://opengrads.org.

The oceanic South Atlantic Convergence Zone (SACZ)^1^ has played a major role during South America’s 2021/2022 summer rainy season, being responsible for more than 80% of the precipitation in some regions of Southeastern Brazil (SEB) and over 90% in some regions in the Southwestern Atlantic Ocean (SWA) as shown in Fig. 1. Oceanic SACZ is an atmospheric system that occurs in South America’s warm season from November to March^2–6^. However, SACZ has this “oceanic” denomination because of its northwest/southeast orientation and its extension over the SWA^1,7–10^. One of the main mechanisms contributing to the SACZ configurations is the so-called South American Monsoon System (SAMS)^11,12^, which favors the moisture, coming from the Tropical Atlantic Ocean, from northern Brazil and the Amazon region to Central and Southeast South America ^2–5,13,14^.

SAMS reaches its most intense phase during the austral summer months (December to February, DJF) and SACZ is one of the most important components of this period^4,11^. DJF climatology exhibits the well-known SACZ configuration with a pronounced and persistent northwest-southeast oriented cloudiness band (areas with Outgoing Longwave Radiation (OLR) below 230 Wm^−2^ in Fig. 1a), explaining the occurrence of more than 50% of annual rainfall in most of the Brazilian southeast and center-west regions, and the southern portions of the Amazon and Northeast Brazil (areas within the blue hatched lines in Fig. 1a). Recent studies have documented the establishment of the oceanic SACZ, due to its extension over the SWA^1,7–10^. Considering the interannual variability of DJF precipitation, particularly in the key regions of the SACZ (continental and oceanic areas, see red boxes in Fig. 1a), the 2021/22 summer displayed the most extreme values in the last decade, whose patterns were associated with the occurrence of seven oceanic SACZ events (Fig. 1b). The composite satellite images for these seven events (Fig. 1c) reveal the dimension of the meteorological phenomenon over South America, investigated in this study.

During the late austral spring and summer seasons, the SACZ occurrence was critical in determining the rainy season quality^5,14^ of SEB, and consequently its climate. Along with low-level jet^15^ that occasionally develops as a core of high-speed flows at the eastern side of the Andes and transports Amazonian humidity to the south/southeastwards of Brazil. The oceanic SACZ serves as an Atmospheric River (AR)^12^ to South America, connecting the Amazon region to the SWA, and transporting moisture from that region to the southeastern part of Brazil. This mechanism has been seldom studied, and in this study, we improve our knowledge about this interesting phenomenon. The atmosphere over the Amazon region can be defined as an “aerial lake of moisture”^12^ which will feed humidity transport to the south/southeast of South America, during the late austral spring and summer seasons. In addition to the scientific world, this subject fascinated the internationally renowned photojournalist *Sebastião Salgado*. Through his lens, he captured amazing pictures of clouds, from the Amazon. They start small early in the morning and grow during the day, generating large convective clouds. This set of clouds will form the ARs, and here is where art meets science in his beautiful photographic “*Amazonia*” exhibition^16^. In his exhibition, Salgado defines the Amazon region as “*Rio Verde*” (Green River), for its ability to provide moisture to the atmosphere, forming a large aerial lake^12^.

The oceanic SACZ is an atmospheric system responsible for producing a sea surface cooling underneath it, and southwestward of it^1,7,8^ where a low-level atmospheric cyclonic vortex is also formed over SWA^1,7,8^. The mechanisms by which this cooling occurs are a complex combination of downward short-wave radiation being attenuated by the cloud cover belt (thermodynamic process)^1^ in conjunction with the reduction of horizontal heat advection in the Brazil Current (BC) region caused by surface winds (a dynamic mechanism)^1^. These cooling mechanisms will be discussed later in this study.

**Figure 2:**
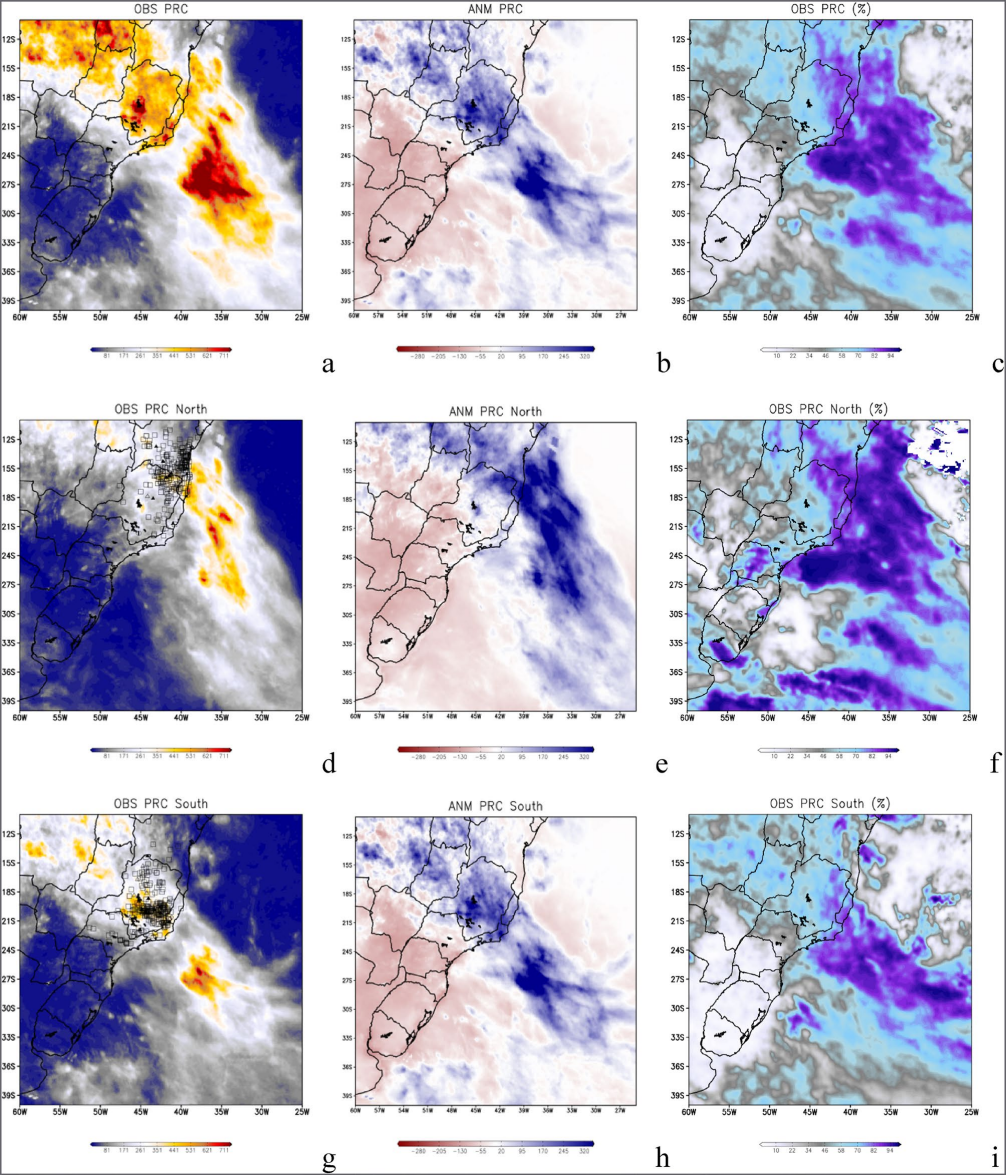
Satellite-derived accumulated (**a**) and anomalies (**b**) of precipitation (mm) over the seven oceanic South Atlantic Convergence Zone (SACZ) cases occurred during the South America summer of 2021/2022. Percentage of accumulated precipitation (**c**) from the seven oceanic SACZ compared to the accumulated climatological precipitation for the summer season (December, January, and February). Figures (**d–f**) are the same as the previous ones but for the cases where the oceanic SACZ were anchored towards the north in December 2021, and (**g–i**) to the south in January and February 2022. Grid Analysis and Display System (GrADS), Version 2.2.1.oga.1. http://opengrads.org.

The 2021/2022 summer was considered an anomalous wet season by official climate monitoring Brazilian institutes. This is attributed to the occurrence of seven cases of intense oceanic SACZ (Table 1), and to the precipitation anomaly charts (Fig. 2) which are discussed later in this study. That summer, according to the National Center for Monitoring and Early Warning of Natural Disasters (CEMADEN)^17^ there were 194 geological hazard events, out of the 574 issued alerts to SEB by CEMADEN (Table 2). These are occurrences associated with landslides on slopes. During the same period, there were also 262 records of flood events, out of the 660 issued alerts. It is worth noting that many of these extreme events and natural disasters have their consequences increased due to the influence of topographic relief. The most affected regions are mountainous with very uneven relief, which in many cases increases the volume of precipitation (Fig. 1c) and consequently amplifies the impacts of landslides and floods, as shown in Fig. 1d.

**Table 1:** Oceanic South Atlantic Convergence Zone (SACZ) characteristics.

Case	Start	End	Duration in days	Position
1st	2021/11/30	2021/12/04	5	North
2nd	2021/12/06	2021/12/10	5	North
3rd	2021/12/23	2022/01/02	10	North
4th	2022/01/05	2022/01/11	7	South
5th	2022/01/29	2022/02/02	5	South
6th	2022/02/07	2022/02/14	8	South
7th	2022/02/16	2022/02/21	6	South

The first column is the case number, and the second and third columns are the start and end date of cases. The fourth column is the oceanic SACZ length in days The last column indicates the SACZ position.

**Table 2:** Number of “Alerts and Events” that occurred in Southeast Brazil during the summer of 2021/2022, issued by the National Center for Monitoring and Early Warning of Natural Disasters (CEMADEN) in www.cemaden.gov.br.

Month	Geological		Hydrological	
	Alert	Events	Alert	Events
December 2021	118	34	206	69
January 2022	252	106	214	118
February 2022	204	54	240	75
Total	574	194	660	262

Some of these anomalous wet season consequences could be verified in the form of floods, landslides, and loss of human lives as reported in the press (https://www.bbc.com/news/world-latin-america-60197978). The SEB is highly populated and climate affect human safety, water supply, food production, and energy generation to this region and others in Brazil. The material damages and economic losses reported by the municipalities to the National Secretariat for Protection and Civil Defense, related to precipitation extremes, corresponded to BRL 2.4 billion in the Bahia and BRL 4.3 billion in Minas Gerais, according to Integrated Disaster Information System data provided by (S2id) (https://s2id.mi.gov.br/) and shown in Table 3. The greatest losses occurred in the 3rd and 4th cases of SACZ, including the turn of the year 2021/2022. In addition to presenting intense precipitations on consecutive days, those cases increased the accumulated rainfall already produced by the previous cases. This increased the moisture saturation in the soil and the volume of water in the rivers, and therefore contributed to major landslides and floods in the states of Bahia and Minas Gerais. These numbers may still be higher, as many municipalities failed to report if they have the resources to cover damages to the national civil defense. By the end of the whole rainy season, 148 human losses were reported, 195,038 homeless/displaced, and BRL 15.9 billion in material damages and economic losses in the states of the Southeast Region of Brazil, according to S2id. Paying attention to the fact that these losses are related to the severity of the precipitation events, but also to the vulnerability and susceptibility of the population living in the areas with potential risks of landslides or floods. Undoubtedly, there is no price that can be associated with the loss of human life that occurred in this period. However, the material damages with losses of family housing provoked a reaction in which the Brazilian Senate approved the Provisional Measure (PM) 1.092/2021, which allocated BRL 700 million to municipalities affected by the rains in December 2021 and January 2022. The funds were intended to cover food distribution expenses and to structure the service network of the Unified Social Assistance System for homeless or displaced people, according to S2id.

**Table 3:** Material damages and economic losses reported by municipalities during South Atlantic Convergence Zone (SACZ) cases, in Brazilian Real (BRL) currency.

Case	Bahia	Minas Gerais	Espírito Santo	São Paulo	Rio de Janeiro
1st	8.6 Mi	231.000			
2nd	435.9 Mi	164.8 Mi			
3rd	1.8 Bi	768.1 Mi	3.5 Mi		5.1 Mi
4th	18.9 Mi	5.5 Bi	3.6 Mi	45.4 Mi	67.3 Mi
5th		36.6 Mi	2.7 Mi	256.4 Mi	829.000
6th		60.9 Mi			68.7 Mi
7th		29.3 Mi	50.3 Mi		9.0 Mi

*Source* S2id (https://s2id.mi.gov.br/).

With the above information in mind, our study addresses the primary natural causes that produced the anomalous summer rainy season of 2021/2022 in Southeast Brazil. This rainy season was unique and rare. We highlighted the main effects of coupling and interaction between the waters of the surface layer of the SWA and the lower atmosphere. In this context, it is shown that the oceanic SACZ is characterized as AR and produced an extremely anomalous amount of precipitation in Southeast Brazil. It is also shown how marine atmospheric boundary layer (MABL) stability is locally modulated by the sea surface temperature (SST) that lies just below it. Up until now, this important mechanism has not been properly highlighted in the literature before this study.

## Results

Following our results, we document and discuss the main characteristics of this abnormally wet rainy season that occurred in Southeast Brazil, which was caused by the occurrence of seven oceanic SACZ events, as listed in Table 1. During the summer of 2021/2022, the highest number of oceanic SACZ was recorded since the summer of 2009/2010 (Fig. 1b, last column). We also show how the oceanic SACZ events are characterized as ARs that transport a very large volume of moisture from the Amazon region, passing through SEB and extending to the SWA. Our analysis also shows the impact of the atmosphere at low levels of the ocean surface currents and ocean surface temperature as well as the ocean feedback onto the atmosphere’s lower levels. This study ends by showing in situ data collected from the *Cryosphere 1* buoy during the first four events of oceanic SACZ. It shows that, even with oceanic cooling caused by the presence of the oceanic SACZ, the ocean surface is still warmer than the overlying air, thus making the ocean a source of energy, moisture, and instability for the lower atmosphere.

### Abnormally wet rainy season

The summer of 2021/2022 in South America was considered an abnormally wet rainy season because the total amount of precipitation exceeding 650 mm in Southeast Brazil as shown in Fig. 2a. The main cause of this wet summer was the occurrence of seven cases of oceanic SACZ during this 3-month period (Table 1) as shown by its average in Fig. 2a–c. This total amount value caused an excess of precipitation compared to historic climatological values, producing 300 mm of positive anomalies of precipitation, as shown in Fig. 2b over SEB and south of Northeast Brazil (NEB) on the continental side. Considering the SWA, over the oceanic side, the total amount of precipitation is even higher, more than 750 mm with anomalies over 350 mm. This is seen when we consider the months of December 2021, January, and February 2022, as shown by the satellite precipitation measurements in Fig. 2. In this context, the oceanic SACZ cases were responsible for more than 80% of the precipitation registered on both continental and oceanic regions, where the SACZ was active during this summer season. It should also be noted that much higher values of precipitation are evident over the ocean, including much more extensive areas, compared to those seen over the continental region (Fig. 2a,b).

The SAMS and SACZ exhibit high spatial–temporal variabilities^11^ that derive from the complex interactions and feedback between the ocean, land surface, and biosphere. In the same summer season cases, where the SACZ anchors further north of the SEB or further south, can be verified. A similar latitudinal SACZ variability was seen in previous studies^18^. There is yet no standard behavior defined in the literature for the continental or oceanic SACZ. When this system becomes more established over the continental region with little or almost no convective activity over the ocean, it is called a continental SACZ. However, when there is highly intense convective activity also occurring over the SWA, it is called oceanic SACZ.

The oceanic SACZ can establish itself in positions further north of SEB, even reaching the southern region of NEB (south of Bahia state) as it can also establish itself further south of SEB, and in this case, reaching the states of São Paulo (SP), Rio de Janeiro (RJ), Minas Gerais (MG) and Espírito Santo (ES). However, there is still no consensus regarding the causes that determine the SACZ’s northern or southern positioning^18^ on the SEB region. During this summer season in our analysis (Table 1), three cases were located further north during spanning the first month while the other subsequent four SACZ cases anchored further south. The feature that uniquely highlights this summer as an anomalous season is the commonality among these seven cases that all of them were cases of oceanic SACZ. This is a unique situation, considering that this is not a common occurrence during a single rainy season. However, what is evident is that during typical summer seasons there is not just one type of SACZ case, rather they are randomly configured in oceanic or continental cases^8^.

The individual SACZ cases were first analyzed and then divided into two groups. One for cases 1 to 3, as shown in Table 1, hereafter referred to as “northern cases” and a second group with the remaining four cases, from 4 to 7 (Table 1) hereafter referred to as “southern cases”. This procedure was adopted to avoid diminishing the SACZ imprint on the average composite fields as they presented spatial variability during the whole summer season.

The northern cases of oceanic SACZ, Fig. 2d–f, have caused large amounts of precipitation over the continent, reaching 500 mm to the south of Bahia and north of Minas Gerais (Fig. 2d) considering the total number of days that the system has remained active. This total precipitation caused positive anomalies larger than 250 mm, compared to the climatological values (Fig. 2e). The amount of precipitation produced by the northern cases of SACZ was greater than 80% of the expected precipitation for the entire month of December 2021 (Fig. 2f).

The southern cases of oceanic SACZ, have caused a larger impact on the central and south regions of Minas Gerais, Rio de Janeiro, Goiás, south of Mato Grosso, and north of São Paulo states as seen in Fig. 2g, and producing amounts of precipitation larger than 500 mm in some of these states. However, extremes of precipitation are also seen over the Atlantic Ocean, with values larger than 700 mm. These extremes of precipitation caused positive anomalies of precipitation when the values over the continent (SWA) were larger than 250 mm and (over 350 mm), respectively. The continental precipitation generated by the southern cases of oceanic SACZ was greater than 80% of the total precipitation expected for both months of January and February 2022. However, over the ocean, this value exceeded 90% for an extensive area, as shown in Fig. 2i.

It is worth mentioning that this total precipitation, although high in accumulation, refers only to the days on which the SACZ cases occurred. Thus, indicating the importance of this phenomenon during the studied period.

### Oceanic South Atlantic Convergence Zone, Atmospheric River, and moisture transport

**Figure 3:**
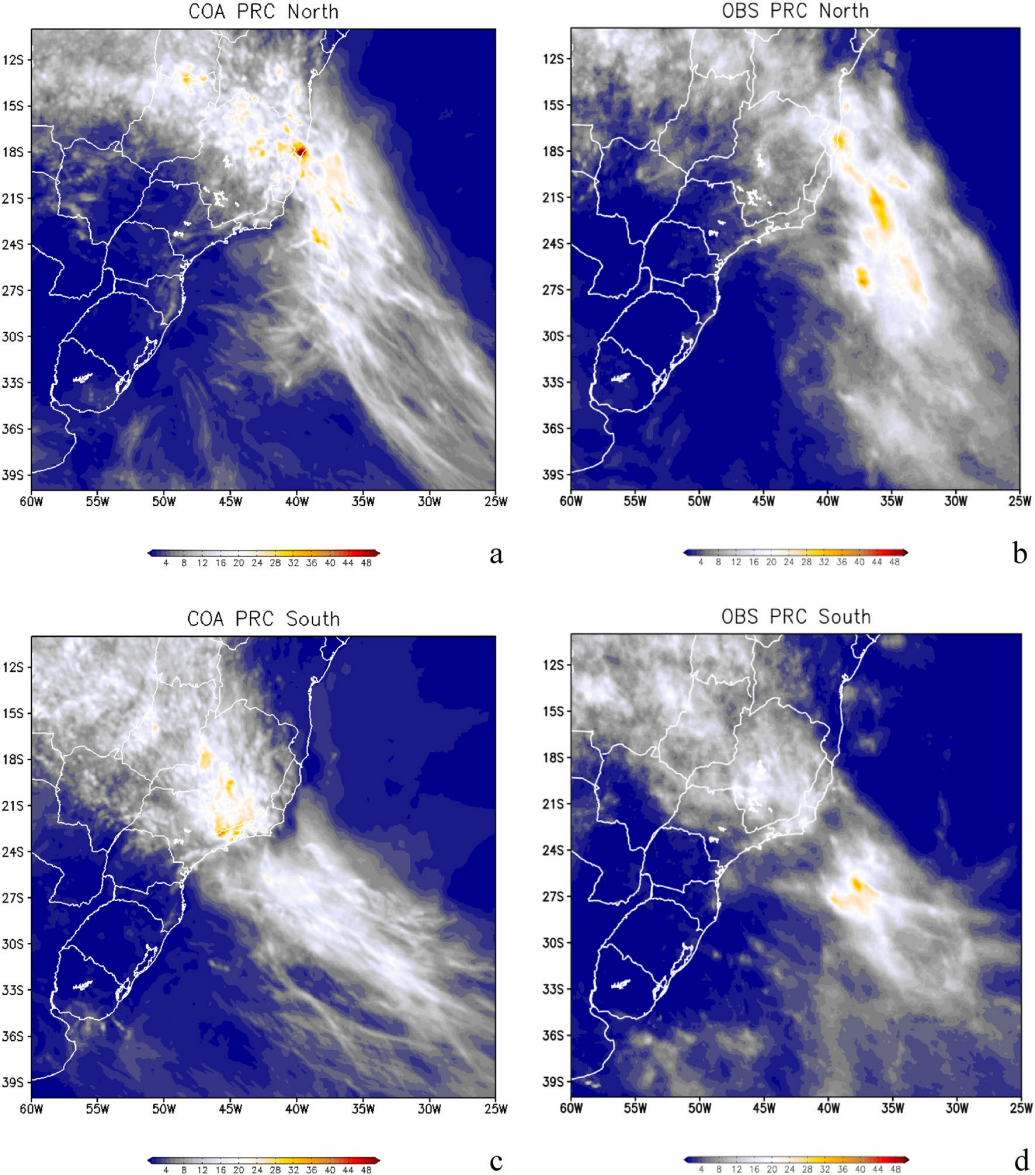
Simulated precipitation (**a**) and observed precipitation (**b**) for the three cases where the oceanic South Atlantic Convergence Zone (SACZ) were anchored further north. Panels (**c,d**) are for the four oceanic SACZ cases when they were anchored further south. Precipitation is in mm day^−1^. Grid Analysis and Display System (GrADS), Version 2.2.1.oga.1. http://opengrads.org.

In this section, we present the set of oceanic SACZ simulations analyzing the main spatial characteristics. First, we analyze and show that our set of simulations can reproduce the expected precipitation patterns of oceanic SACZ when compared against satellite data. Finally, an analysis is performed to evaluate the dynamics and thermodynamics of the simulated oceanic SACZ in SWA. We analyze simulations made by our regional coupled ocean–atmosphere model system (COAWST) (see “Methodology” section for a full COAWST setup description). The oceanic SACZ’s cloud band position and intensity can be inferred from the precipitation (mm day^−1^) as shown in Fig. 3a,c. The model experiments were able to adequately simulate the main features of the oceanic SACZ, which are also observed in the satellite-derived precipitation data. The elongated feature of the precipitation band that extends from the northwest (originated in the Amazon, but not represented in our domain), passing through the SEB region, and going towards the Southwest Atlantic region, is present in all the simulations. However, it should be noted that the model slightly underestimates the extremes. The oceanic SACZ cases are adequately simulated, considering that the precipitation field is one of the most difficult to simulate with high precision and often needs to be corrected after its simulation ^19^. The satisfactory skill of the model is good and can be seen by comparing both the simulated northern cases (Fig. 3a) with the observed cases of SACZ (Fig. 3b) and the simulated southern cases of SACZ (Fig. 3c) with observed ones (Fig. 3d).

ARs are characterized by having a long spatial extension comprising a very large volume of moisture in the air that extends from the surface to the highest levels of the atmosphere. They are analogous to surface rivers, as they have a large volume of precipitable water. In the case of South America, they arise because of the presence of the SAMS, during the South American summer^12,20^. The moisture coming from the tropical Atlantic is channeled and transported to the Southeast and South of Brazil. There is no doubt that this mechanism plays a fundamental role in the climate of Brazil and of South America ^20^, as well as in the Atlantic Forest and in Brazilian coastal ecosystems such as the salt marshes and mangrove forests^21^.

**Figure 4:**
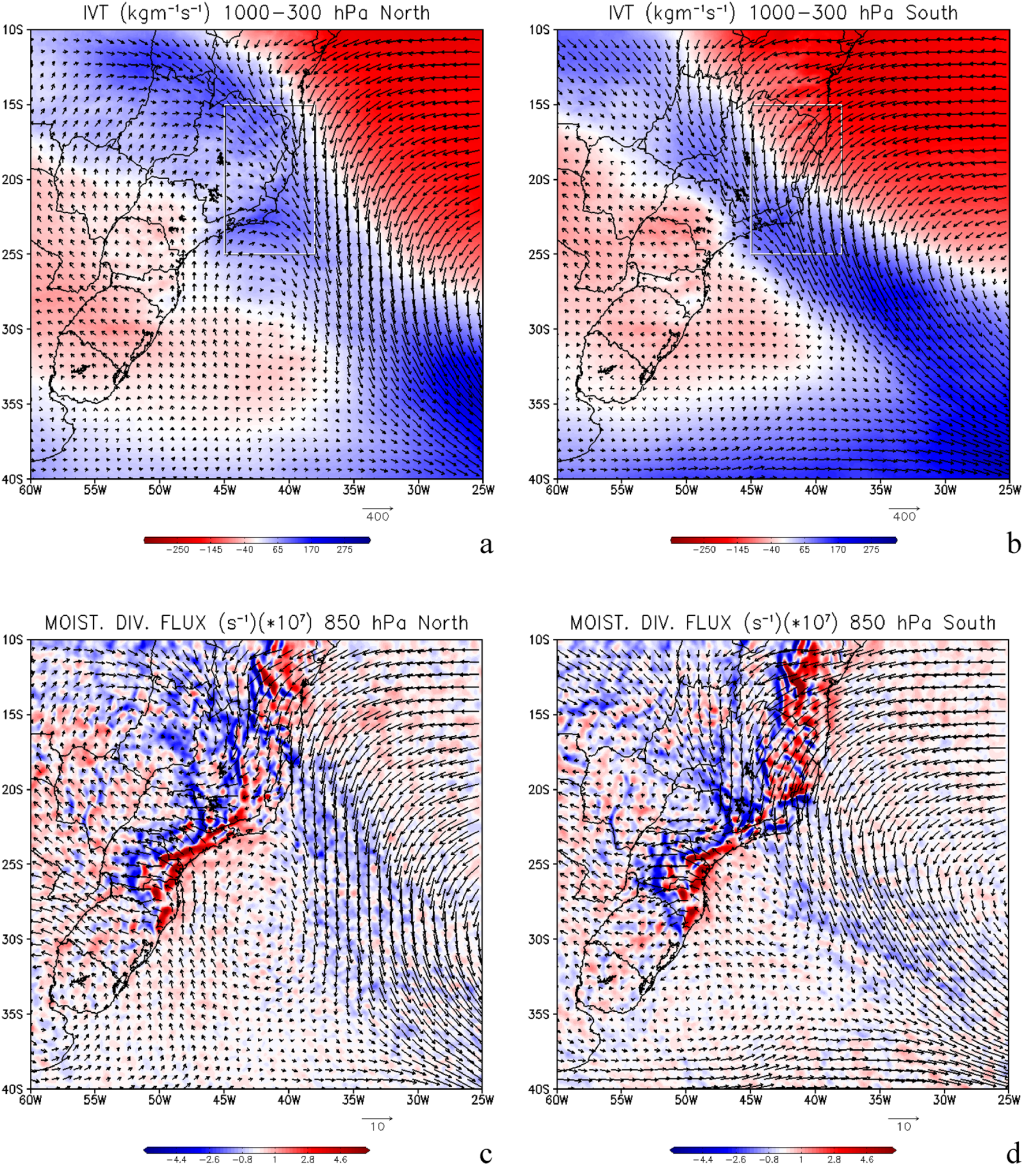

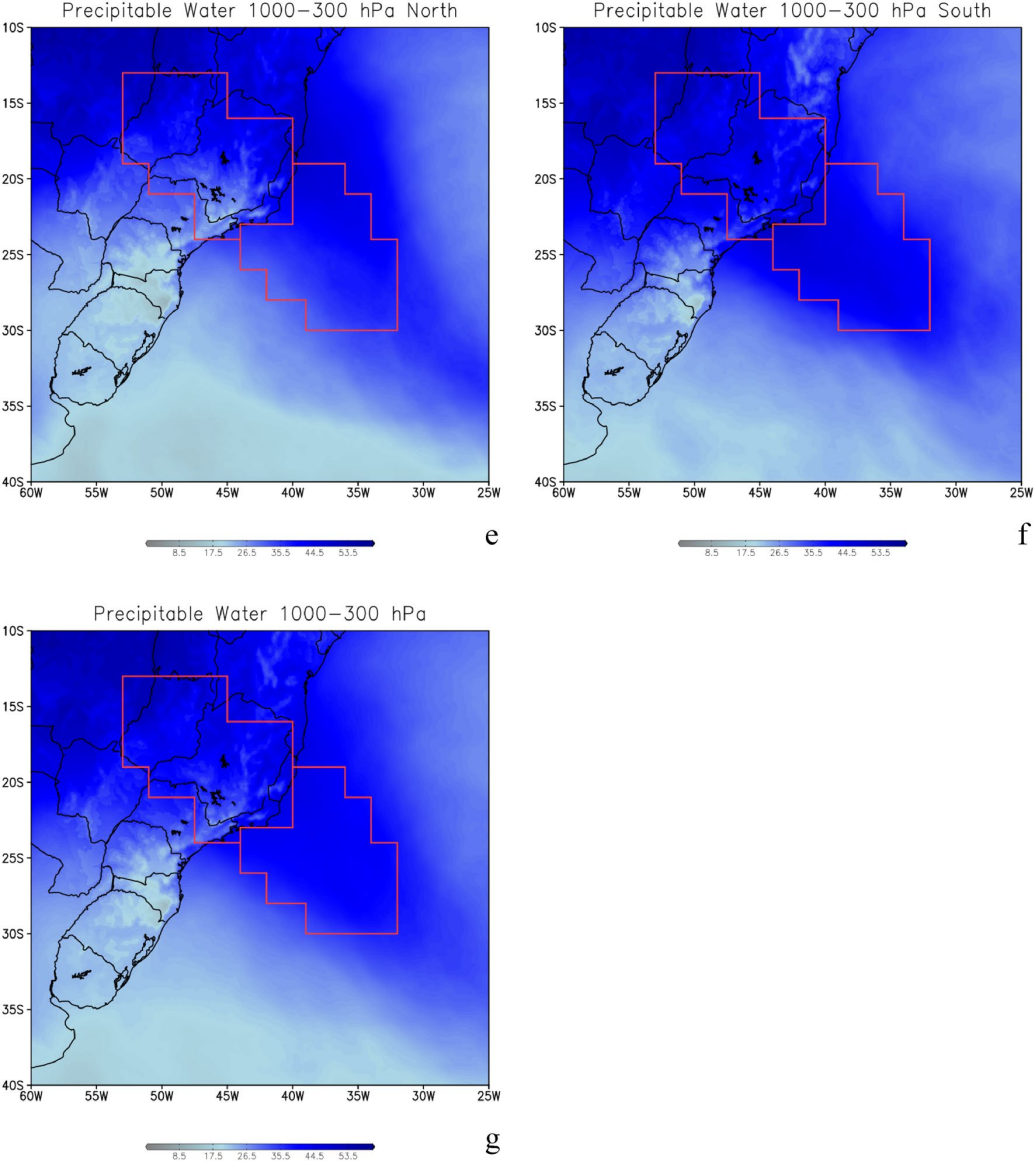
Integrated vapor transport (IVT) from 1000 to 300 hPa for the three cases where the oceanic South Atlantic Convergence Zone (SACZ) were anchored further north (**a**) and for the four oceanic SACZ cases when they were anchored further south (**b**), in kg m^−1^ s^−1^. Moisture divergence flux (shaded) and zonal moisture flux transport (vectors) for the north cases (**c**) and south cases (**d**), in 1 × 10^7^ s^−1^. Precipitable water (PW) vapor from 1000 to 300 hPa for the three cases where the oceanic SACZ were anchored further north (**e**), for the four oceanic SACZ cases where they were anchored further south (**f,g**) for all the seven cases, in mm day^−1^. The red lines represent the areas where the precipitable water was averaged and presented in Table 4. The continental area is over the continent, the oceanic area is over the ocean. The total area considers both areas. Grid Analysis and Display System (GrADS), Version 2.2.1.oga.1. http://opengrads.org.

The simulated moisture transport and produced by the oceanic SACZ is shown in Fig. 4 which depicts the integrated vapor transport (IVT) from 1000 to 300 hPa for the three cases where the oceanic SACZ were anchored further north (Fig. 4a) and for the four oceanic SACZ cases anchored further south (Fig. 4b). We can see a well-defined narrow and elongated corridor of humidity being transported from northwest to southeast, within the troposphere as a typical atmospheric river (Fig. 4). The E, ENE, and NW winds indicate the western branch of the South Atlantic Subtropical High (SASH) contribution to the oceanic SACZ, as shown in Fig. 4. Another striking feature of the oceanic SACZ cases, which appear well defined in our results, even with vertically integrated winds, is the surface cyclonic circulation, configuring the Cyclonic Vortex Southwest of SACZ (CVSS)^1,7,8^. These dynamic features appear for both northerly and southerly located systems. However, this cyclonic circulation that appears in these figures will be discussed later in “Oceanic South Atlantic Convergence Zone and surface circulation” section while discussing surface atmospheric circulation. Moisture divergence flux (shaded) with winds superimposed (vectors) for the northern cases is shown in Fig. 4c and for southern cases in Fig. 4d. The negative values indicate moisture convergence at 850 hPa. This atmospheric variable is evaluated together with precipitation (Figs. 1a, 2) and corroborates the information about the source of humidity to SACZ, which comes from the Amazon region (northwest) with local contribution from the southwest Atlantic Ocean on northern (Fig. 4c) and southern (Fig. 4d) cases.

The AR analysis is complemented by our precipitable water (PW) calculation, evaluated as the specific humidity integrated from the surface up to 300 hPa. The total water vapor content in a vertical atmospheric column per unit area is equivalent to the PW, which is, the amount of water potentially available in the atmosphere for precipitation is shown in Fig. 4, for northern (Fig. 4e) and southern (Fig. 4f) cases. This analysis reveals the AR extension from the Amazon region to SWA and the meridional displacement for the northern (Fig. 4e) and southern (Fig. 4f) cases. A striking feature is revealed in Table 4, which shows the sum area of PW (in mm day^−1^) over each area indicated in Fig. 4e–g. The areas are over the continent, and over the ocean, and the total area includes both, for the oceanic SACZ cases. The PW average brings the information that the amount of water in the atmosphere in each of these areas is very large. The amount is greater in the southern cases for both regions over the continent (slightly larger) and over the ocean, which have a direct influence on the Amazon region, with 433.5 mm day^−1^ for the continental region and 502.5 mm day^−1^ for the oceanic region. As we see in regions further north, this amount is still very large, with a reduction of approximately 13.7% over the continent and 7.5% over the oceanic region, compared to the southern cases. The larger amount of PW is also found in the southern cases when the total value (in both areas marked by the red line in Fig. 4e–g is 938.8 mm day^−1^ as shown in Table 4.

**Table 4:** Average of precipitable water (PW) vapor in mm day^−1^ over each area indicated in Fig. 4e–g.

Area	Continent	Ocean	Total
North	373.7	467.4	841.1
South	433.5	505.2	938.8
Average	403.6	486.3	889.9

The areas are over the continent, the ocean, and the total area that considers the two previous ones, for the oceanic SACZ cases.

### Oceanic South Atlantic Convergence Zone and surface circulation

**Figure 5:**
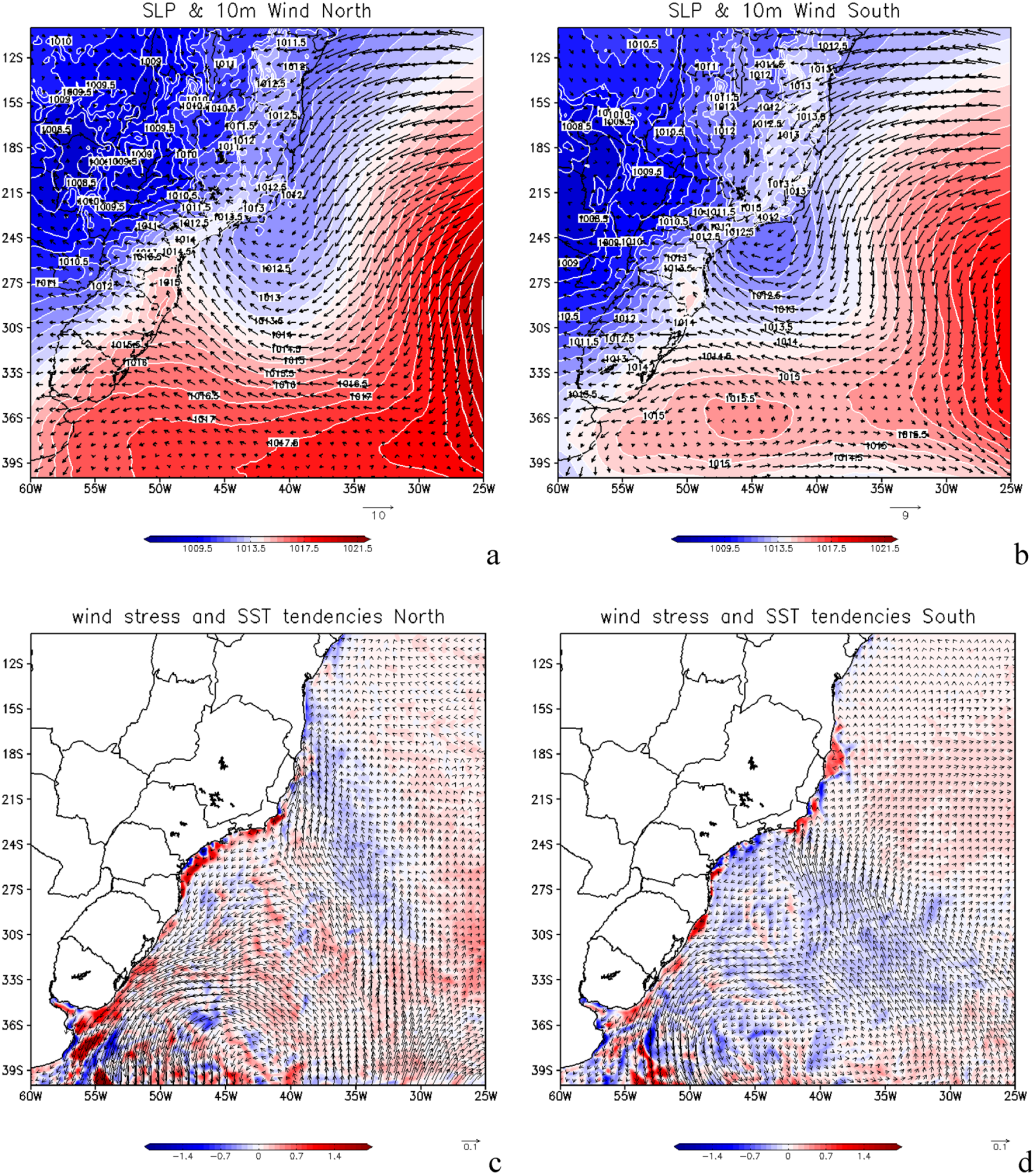
Winds at 10 m height superimposed on Sea Level Pressure (SLP), in m s^−1^ and hPa, respectively, for the three cases where the oceanic South Atlantic Convergence Zone (SACZ) were anchored further north (**a**) and for the four oceanic SACZ cases when they were anchored further south (**b**). Wind stress tendency (N m^−2^) superimposed on Sea Surface Temperature (SST) tendency (°C) for the further north cases (**c**) and for the further south (**d**). Grid Analysis and Display System (GrADS), Version 2.2.1.oga.1. http://opengrads.org.

Here, we analyze the sea level pressure (SLP) and associated surface wind field at 10 m height during oceanic SACZ events (Fig. 5a,b). The western branch of South Atlantic Semipermanent High (SASH) is indicated in these figures by the E and ENE winds, for both northern (Fig. 5a) and southern (Fig. 5b) cases. Near the coast around 24° S is the Southern Brazilian Continental Shelf (SBCS), and around 24° S a low pressure region (trough) with low-level cyclonic circulation is seen, configuring the CVSS^1,7,8^. The CVSS also shows a meridional displacement when comparing the northern (Fig. 5a) with the southern (Fig. 5b) cases.

The sea surface cooling underneath the SACZ position is seen through the analysis of the SST trend shown in Fig. 5a,b. The cooling is a consequence of the lower-level atmospheric circulation that has its intensity reduced while SACZ episodes are occurring. This is seen from the wind stress trend also shown in Fig. 5c when oceanic SACZ is displaced northward and in Fig. 5d when oceanic SACZ is displaced southward. This analysis of the cooling mechanism is further complemented in “Oceanic South Atlantic Convergence Zone and oceanic volume transport” section.

### Oceanic South Atlantic Convergence Zone and oceanic volume transport

The mechanisms by which the SWA is surface-cooled have been discussed in the literature^1,7,22,23^. This is also associated with SACZ negative feedback^23^. One of the causes is potentially the thermodynamic mechanism, which is due to the blocking of shortwave radiation from the sun, by the SACZ cloud cover. However, a second mechanism, attributed to oceanic dynamics could also exist^1,22,23^. In some studies, it is shown to be associated with oceanic upwelling, caused by the surface winds, and the consequent horizontal flux divergence at the surface. Thus, the configuration of surface winds that favor upwelling are present, but they are not the main driver for cooling^1^. This vertical cooling is not expressed because, in this region, the sub-surface waters in the first 50 m are not considerably colder compared to the surface waters^1^.

**Figure 6:**
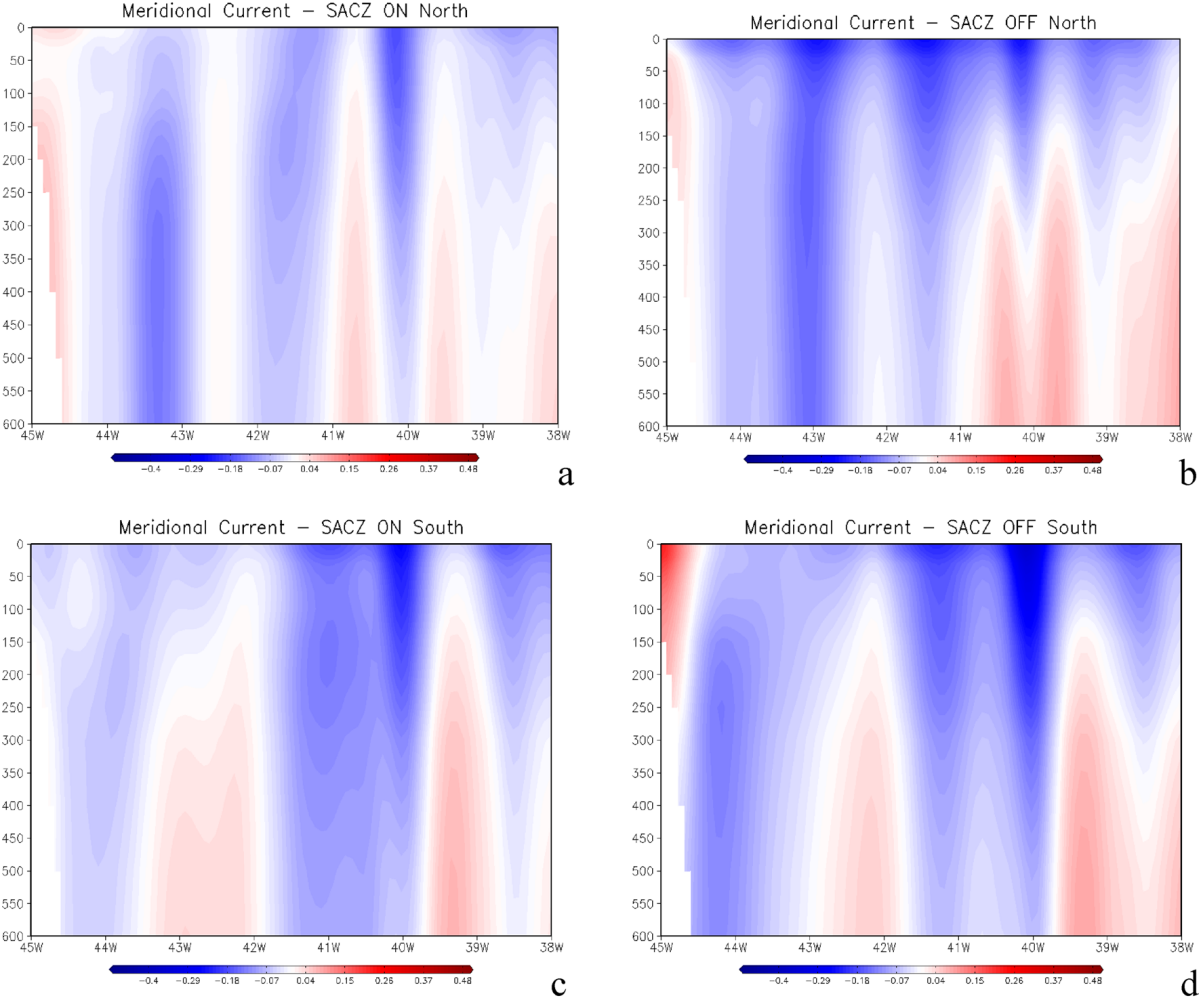
Meridional current (v) profile in m s^−1^ averaged over a latitudinal band extending from 15° to 25° S, as shown in Fig. 4a,b, for the three cases where the oceanic South Atlantic Convergence Zone (SACZ) were anchored further north (**a**) and the day before oceanic SACZ occurrence (SACZ off) in (**b**). Panel (**c**) is for the four oceanic SACZ cases when they were anchored further south and (**d**) is for the day before oceanic SACZ occurrence. The respective values of volume transport calculations for each panel are (**a**) − 8.31 Sv, (**b**) − 11.96 Sv, (**c**) − 10.61 Sv, (**d**) − 12.71 Sv, where 1 Sv = 10^6^ m^3^ s^−1^). Grid Analysis and Display System (GrADS), Version 2.2.1.oga.1. http://opengrads.org.

However, there is also a modulation caused by surface winds in the surface oceanic current system, which consequently alters the horizontal transport of heat in the region near the location of the oceanic SACZ^1,8^. In the presence of SACZ, the transport of heat from the north is reduced, mainly caused by the decrease in the speed of the oceanic currents from the north and by the reduction of the ocean volume transport, as shown in Fig. 6 and also reported in the literature^1^.

This is also seen in our results, in the northern cases, where the average total volume transported over a latitudinal band extending from 15° to 25° S, (red box in Fig. 4a,b) was − 8.31 Sv (1 Sv = 10^6^ m^3^ s^−1^) when the currents are weaker as seen in Fig. 6a. Whereas before the northernmost episodes occurred, considering the most intense currents in these cases, the associated transport was considerably larger with values reaching − 11.96 Sv, as shown in Fig. 6b. It is important to remember that negative values indicate the direction of transport from north to south. This same behavior is observed for the cases of SACZ when they are positioned, further south. There is a reduction in the volume of transported water (− 10.61 Sv) when the SACZ was active (Fig. 6c) when compared to the previous period of SACZ occurrence when the transport reached − 12.71 Sv (Fig. 6d).

### Oceanic South Atlantic Convergence Zone and the local marine atmospheric boundary layer stability

**Figure 7:**
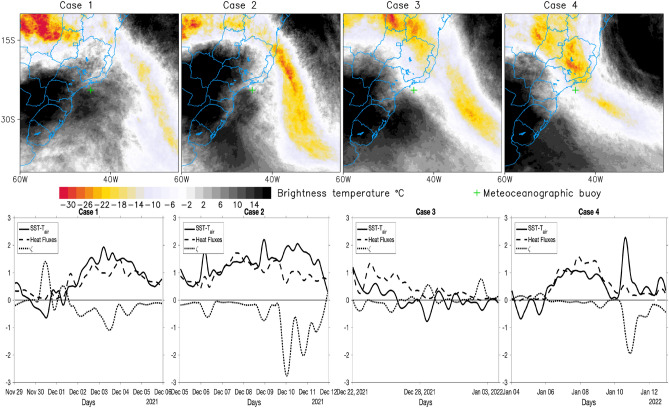
Satellite brightness temperature composites (°C) (top panel) for the SACZ cases 1 to 4 and corresponding in situ buoy measurements (bottom panel) of Heat Fluxes (Ql + Qh)/100 W m^−2^ and SST-T_air_ (°C) and Monin–Obukhov (ξ = Z/L) stability parameters (dimensionless). The green cross in the satellite images indicates the position of the Cryosphere meteoceanographic buoy. Grid Analysis and Display System (GrADS), Version 2.2.1.oga.1. http://opengrads.org. MATLAB, Version 9.1.0.441655 (R2016b). https://www.mathworks.com.

The meteoceanographic buoy, *Cryosphere 1* recorded the oceanic SACZ, during the first four cases, as shown in the top panels of the satellite brightness temperature composites (°C) (indicated by a green cross in Fig. 7). The presence of oceanic SACZ produces cooling of the ocean surface, both by cloud cover which is defined as the thermodynamic mechanism^8^, and by decreasing heat transport to the south by the weakening of the current system as shown in Fig. 6, which is defined as the dynamic mechanism^1^. However, the interesting information that our meteoceanographic observations from *Cryosphere 1* have revealed is that even with the sea surface being colder, it is still warmer than the air. Thus, the ocean still acts as a source of heat for the atmosphere, even though it is colder than before the SACZ has been established. Thus, the MABL has unstable conditions. This is seen by the heat fluxes calculated from the bulk formulas, by the near-surface MABL stability parameters computed from SST-T_air,_ and by the ζ^24–27^ as shown in the bottom panels of Fig. 7, for 1st to 4th cases. Positive SST anomalies would induce changes in the MABL static stability. In this case, the air buoyancy and turbulence would increase over warmer waters thus reducing vertical wind shear at the boundary layer and generating stronger winds at the sea surface. A similar situation has already been reported in the literature in regions where intense oceanic frontal gradients occur, both caused by oceanographic fronts^24–26^ and due to the oceanic mesoscale processes^28,29^. The buoyancy of the air parcel overlying the ocean and the air turbulence confined to the MABL are both increased over warmer waters. This is a clear indication of the local modulation of SST over MABL. Consequently, vertical wind shear at the boundary layer is reduced, generating stronger winds at the sea surface. We believe that in cases of oceanic SACZ, this is the first time that this mechanism is being verified and registered. In other words, there are indications that the ocean, in addition to being dynamically and thermodynamically modulated by the atmosphere, is also providing a feedback process to MABL, and consequently contributing to the maintenance of the air instability caused by SACZ. The Cryosphere 1 buoy was near the southwestern edge of the SACZ, in cases 1 to 3. It is noteworthy that after case 4 the buoy drifted from its original position, but even so, it made precious records before being removed from the water.

## Final remarks

There is still much to be studied and understood about the impact, modulation, and feedback mechanisms of the oceanic SACZ and its interaction with the ocean as well as understanding this phenomenon’s behavior in recent years, assuming that the climate is changing. Our study of the 2021/2022 rainy season was motivated by its unique occurrence of seven cases of oceanic SACZ, which had an extreme impact on the society of Southeast Brazil.

Approximately 90% of the total precipitation observed in the rainy season in Southeast Brazil, was derived from the occurrence of seven cases of oceanic SACZ. The extreme excess of precipitation that occurred, caused a lot of economic losses and great damage to Brazilian society. Several cases of flooding were recorded in urban regions in the Southeast Brazil, in addition to landslides in regions on hillsides.

Little information is available in the literature about the dynamic and thermodynamic characteristics of the oceanic SACZ. Here we show an interesting and relevant aspect of this phenomenon, which are the characteristics of an Atmospheric River. The Amazon is considered as a natural reservoir of water, supplied both by the soil and by atmospheric circulation. The moisture coming from the Tropical Atlantic oceanic region is advected to the top of the forest. The junction of both sources of moisture when the South American monsoon regime is active and the oceanic SACZ is established channels in the form of an AR passing through Midwest and Southeast Brazil, and the Southwest Atlantic. The total amount of water released can approximately reach 20 Gt (or 20 trillion liters) in 1 day^30^. This discharge is comparable to the ones of the Amazon River. Consequently, the volume of precipitable water transported by an AR and precipitated over the Southeast region of Brazil will also be large, as observed and highlighted in this study.

The southward oceanic volume transport is also reduced when oceanic SACZ is on. Its reduction is also consequently seen in heat transport from the tropical area when oceanic SACZ exists, corroborating previous results found in the literature^1^. The Southwest Atlantic is a complex region in terms of oceanographic phenomena. The coastal region is quite susceptible to the upwelling of cooler and subsurface waters, such as the outcrop of the South Atlantic Central Water (SACW) mass in the region of Cabo Frio, in the state of Rio de Janeiro. Towards the offshore region, where the oceanic SACZ is positioned, the SACW is deeper, and the dynamic conditions favor the upwelling caused by surface winds that, however, do not cool the oceanic surface^1^. In this case, the meridional weakened heat transport coming from the north may be reducing the heat content in this region and consequently decreasing the SST.

Our knowledge gained from this study concludes with the unprecedented results of how the MABL is locally modulated by the SST that lies just below it. This is the first time this issue has been demonstrated in the literature, showing that even though the ocean is colder than before oceanic SACZ was established, it is still warmer than the overlying air. Thus, it becomes an active source of heat to the atmosphere and helping the MABL instability process. This result was obtained thanks to the *Cryosphere 1* buoy being present at the location of the first four cases of oceanic SACZ in the summer of 2021/2022.

There is no doubt that these are processes that should be revisited when more in situ data is available. There is still much to be learned about which ocean phenomena are the most important in this ocean surface cooling process and how they function. It is especially important to better understand how large-scale atmospheric systems interact with the Southwest Atlantic when the oceanic SACZ is established, such as the SASH.

## Methodology

### Regional coupled models

In this study we used the Coupled Ocean–Atmosphere–Wave–Sediment Transport (COAWST) Modeling System v 3.7^31^. This modeling system combines four numerical models, one for each: the atmosphere, ocean, ocean waves, and water sediment transport. However, in this study we used only two of them: (i) the atmospheric model which is the Weather Research and Forecasting Model (WRF)^32^; (ii) the Regional Ocean Modeling System (ROMS)^33^ which is the hydrodynamic ocean model. All these models can be actively coupled using the Model Coupling Toolkit v 2.6.0 (MCT). COAWST is an open-source system that is based on individual open-source models^31^.

This system allows regional simulations of synoptic-scale thermodynamic and dynamic systems for atmospheric^34,35^, oceanic^36^, and coupled^1^ phenomena. When regionalized, this system can produce more detailed simulations, allowing the assessment of its effects at regional scales^1,34–36^. This was the case of the simulation made for the hybrid hurricane “Catarina”; the first recorded in the South Atlantic, that landed in Santa Catarina^35,37^, and also in the cases studied of oceanic SACZ^1^.

All the physical parameterizations used here for the ocean model ROMS, are the same as those used in a previous study^1^, and for the sake of brevity, we will not reproduce them here. However, a few different choices of atmospheric parametrizations were employed in this study, and the following physical schemes were used to configure the WRF as listed here: (a) the microphysics was the Ferrier scheme, (b) the cumulus convection was the New Tiedtke scheme^38^, (c) Planetary Boundary Layer was the YSU scheme^39^, and (d) radiation for both Longwave and Shortwave was the GFDL scheme^40^.

The surface layer scheme used was MM5^41^ and for the land–atmosphere interactions we used Noah Land Surface Model^42^. It is important to remark that the WRF schemes used in this study were tested and compared to the ones used in a previous study and they showed to be more suitable for the precipitation simulated by WRF, which was a goal in this study. This comparison is not shown here, however, the reader can gauge WRF skill by comparing model precipitation to the satellite observations as shown in Fig. 3.

### Selection of cases and numerical experiments

The selected cases for the study of the oceanic SACZ and the periods of numerical integration were based on the same criteria as those used in previous work made by our team^1,8^. It is based on parameters, such as cloud cover band defined by outgoing longwave radiation (OLR) values lower than 230 W m^−2^, life cycle longer than 4 days, and positioning over the SACZ region. The OLR fields were superimposed on wind fields at 850 hPa, 200 hPa, and vertical velocity at 500 hPa (not shown). From this information, we were able to identify and classify the seven cases (Table 2) as oceanic SACZ, based on their well-defined oceanic configurations.

Seven numerical experiments were carried out, one for each selected case indicated in Table 1. All numerical integrations started the day before the cases were established, and this previous day was excluded from the analysis. Each model starts from its own boundary and initial conditions. After 1.800 s of integration (user selected), the models reach a synchronization point where the fluxes are exchanged between the ocean and atmosphere. The atmospheric domain was configured to match the same oceanic domain, but with a horizontal resolution slightly finer than the oceanic, 6 km, approximately. The model also uses terrain-following vertical-sigma coordinates with 45 levels. The model setup used here can be seen in a previous study^1^ which used the same setup used here and proved to be adequate for this kind of study.

The atmospheric data set used in this study are from the Climate Forecast System reanalysis, version 2 (CFSv2)^43^ and can be found at https://rda.ucar.edu/. They were obtained from the 6-hourly products with 0.5° × 0.5° of spatial resolution. Ocean data are from the eddy-resolving global re-analysis Glorys12v1^44^ that provides daily averaged data with 1/12° of horizontal resolution on a regular latitude–longitude grid and with 50 standard vertical levels. The oceanic model component is the Nucleus for European Modelling of the Ocean (NEMO) platform^45^. It is driven at the surface by European Centre for Medium-Range Weather Forecasts (ECMWF) ERA-Interim atmospheric re-analysis^46^.

### Auxiliary data and fluxes calculations

Auxiliary satellite data were used in this study mainly to diagnose the cases of oceanic SACZ and verify if the model satisfactorily simulated the episodes. This was done for precipitation, for the cloud top brightness temperature, and for the outgoing long-wave radiation emitted into space. The objective was also to verify, in a subjective way, if the coupled model system performed satisfactorily in simulating the oceanic SACZ cases.

The satellite data of OLR used here were obtained from National Oceanic and Atmospheric Administration (NOAA) Daily OLR^47^. The OLR data is retrieved twice daily by Advanced Very High-Resolution Radiometer (AVHRR) scanner that operates in six channels, 3 in the region of visible-near infrared and 3 in thermal infrared. This data set is available as daily values from January 1st, 2002 to the present, 2.5° latitude × 2.5° longitude global grid. The global satellite data of brightness temperature (Tb) is the GridSat^48^ product that provides equal-angle gridded uniform observations every 3 h from 1980 to the present with a spatial resolution of 8 km. The Global Satellite Mapping of Precipitation (GSMaP), version MVK (GSMaP-MVK) is a set of new high-resolution precipitation estimates based on blending passive microwave (PMW) sensors and infrared (IR) radiometer data to produce estimates of precipitation over the globe at a spatial resolution of 0.1° latitude/longitude and hourly temporal resolution^49^. As remarked by many investigators satellite data supply crucial information where in situ data are very sparse or nonexistent, which is the case over the Southwest Atlantic (a major focus of this study) and some parts of the South American continent.

The meteoceanographic buoy, *Cryosphere 1* was moored at 24.4° S and 44.4° W and remained there from November 24, 2021 to January 20, 2022. This buoy is equipped with sensors to measure atmospheric parameters, including air temperature, wind speed and direction, atmospheric pressure at sea level, relative humidity and solar radiation. The ocean parameters are sea surface temperature and salinity. Data is measured every 5 min, hourly averaged and then transmitted in real time using the Iridium satellite system.

The turbulent latent and sensible heat fluxes, and atmospheric stability parameter were calculated using the observed hourly data from *Cryosphere 1* using the well-known, bulk parameterization COARE 3.5^27^. The basis of the parameterization algorithm applied here lies on the Monin–Obukhov similarity theory that considers the flux as constant at the sea surface layer. Ocean–atmosphere interaction studies^24,25,28,50^ carried out in the region of the Brazil-Malvinas Confluence in the SWA have successfully used this methodology to calculate turbulent fluxes.

### Volume transport

Ocean current modulation associated with oceanic SACZ presence was assessed through the vertical profiles of meridional (v) current, which were complemented by the volume transport (VT) calculations and analysis over the area shown in Fig. 6. We performed this calculation, first averaging v over a latitudinal band extending from 15° to 25° S, as shown in the red box in Fig. 4a,b. This area extends from 45° to 38° W and from the surface down to 600 m depth, as shown in Fig. 6. The unit of volume transport is given in Sverdrup (Sv) where 1 Sv = 10^6^ m^3^ s^−1^. The VT is defined here as the sum of all northward/southward transports from the section-width integrated volume flux profile:

$${\int }_{600}^{0}{\int }_{45W}^{38W}v dxdz.$$

## Data Availability

The datasets used and/or analyzed during the current study will be made available from the corresponding author on reasonable request.
